# A tryptophan metabolism-related gene signature predicts prognosis and immune features in cutaneous melanoma

**DOI:** 10.3389/fimmu.2026.1806319

**Published:** 2026-05-19

**Authors:** Lei Zhao, Zhi-Cai Li, Zhou-You Tang, Yu-Tong Yuan, Shan Zhu

**Affiliations:** Plastic Surgery Hospital, Chinese Academy of Medical Sciences and Peking Union Medical College, Beijing, China

**Keywords:** cutaneous melanoma, IDO1, prognostic signature, tryptophan metabolism, tumor immune microenvironment

## Abstract

**Introduction:**

Tryptophan metabolism is known to affect tumor immunity. However, the value of tryptophan metabolism-related genes (TMRGs) in predicting prognosis and reflecting immune status in skin cutaneous melanoma (SKCM) is not yet clear.

**Methods:**

We analyzed transcriptomic and clinical data of 457 patients with SKCM to elucidate the expression patterns of TMRGs and their links with survival and the tumor immune microenvironment. Unsupervised clustering and Cox regression were used to identify prognostic genes and build a TMRG-based risk model, which was then tested in independent cohorts. Immune features were further studied using bulk RNA sequencing and single-cell RNA sequencing data. In addition, functional experiments were performed in melanoma cell lines after *IDO1* knockdown.

**Results:**

The expression of TMRGs was widely altered in SKCM and was related to immune cell infiltration, tumor stemness, mutation features, and metabolic pathway activity. A five-gene risk model, comprising HADHA, GOT2, STAT1, CAT, and IDO1, divided patients into high- and low-risk groups with significantly different overall survival rates, and this model remained an independent predictor even after adjustment for clinicopathological factors. The two risk groups also showed apparent differences in the immune microenvironment, including immune cell composition and immune-related gene expression. Drug sensitivity analysis suggested that the two groups may respond differently to several chemotherapeutic and targeted drugs. *In vitro* experiments showed that IDO1 knockdown altered proliferation, migration, apoptosis, mitochondrial function, and tryptophan metabolism in melanoma cells.

**Discussion:**

These findings suggest that tryptophan metabolism is closely linked to immune heterogeneity and clinical outcomes in patients with SKCM. Moreover, the TMRG-based risk model developed in this study provides complementary prognostic information and a basis for further investigation of metabolism-associated immune regulation in melanoma.

## 1 Introduction

Skin cutaneous melanoma (SKCM) is one of the most aggressive malignancies of the skin and the predominant subtype of melanoma worldwide (1). Despite accounting for a relatively small proportion of skin cancers, SKCM contributes disproportionately to skin cancer-related mortality, owing to its strong metastatic potential (2). Clinically, melanoma progression ranges from localized primary lesions with favorable outcomes to advanced metastatic disease, with limited median survival despite recent therapeutic advances (3).

Complete surgical excision remains the cornerstone of curative treatment in SKCM (4). Patients with advanced or metastatic melanoma show poor outcomes. In recent years, immune checkpoint inhibitors, especially drugs targeting the programmed cell death protein 1 (PD-1) pathway, have improved survival in some patients with SKCM (5). Notably, in individuals harboring BRAF-V600 mutations, combined targeted and immunotherapeutic strategies have extended the median overall survival (OS) duration from less than 1 year to beyond 2 years (6). However, the durable clinical benefit of PD-1 blockade has been observed in only a minority of patients, with objective response rates generally ranging from 20% to 40% (7). Primary resistance and acquired nonresponse remain major clinical challenges, underscoring the urgent need for reliable biomarkers and mechanistic insights that can predict immunotherapy responsiveness.

Tryptophan (Trp) metabolism is considered an important part of immune balance and tumor–immune interaction. In mammals, Trp is mainly broken down through the kynurenine pathway and serotonin pathway. The kynurenine pathway produces several active metabolites, such as kynurenine, kynurenic acid, xanthurenic acid, and quinolinic acid, many of which can affect immune activity in the tumor microenvironment (TME) (8–10).

Abnormal activation of Trp catabolism is usually marked by local depletion of Trp and accumulation of immunosuppressive kynurenine metabolites. These changes may reduce effector T-cell proliferation, promote regulatory T-cell differentiation, and lead to immune tolerance (11, 12). The enzymes driving this process, most notably indoleamine 2,3-dioxygenase 1 (IDO1) and Trp 2,3-dioxygenase (TDO2), are frequently overexpressed or hyperactivated in multiple malignancies, including brain, breast, gastrointestinal, and lung cancers (13–16). By reshaping the metabolic and immune landscapes, dysregulated Trp metabolism could facilitate tumor immune escape and disease progression (17).

Despite the recognized importance of Trp metabolism in cancer biology, its multifaceted role in SKCM remains to be fully elucidated. In particular, the expression patterns, prognostic relevance, and immune regulatory functions of Trp metabolism-related genes (TMRGs) in melanoma have not yet been systematically explored. In addition, how TMRG-related metabolic changes shape the tumor microenvironment and affect treatment response, especially response to immune checkpoint blockade, is still largely unknown.

In this study, we performed an integrative multi-omics analysis to systematically investigate the molecular and clinical characteristics of TMRGs in SKCM. By examining the expression profiles, prognostic significance, immune infiltration characteristics, and associations with therapeutic sensitivity, we aimed to elucidate the contribution of Trp metabolism to melanoma heterogeneity and immune regulation. This study provides additional insights into metabolic–immune interactions in SKCM. Moreover, it may contribute to improved risk assessment and outcome stratification in SKCM.

## 2 Materials and methods

### 2.1 Data collection

A set of 46 genes associated with Trp metabolism was curated from previously published literature; the details are summarized in **Supplementary Table 1** (18). The transcriptomic profiles of SKCM and normal skin tissues were retrieved from the GTEx and TCGA databases using UCSC Xena. The final dataset included data on 457 melanoma and 812 normal skin samples. Two independent cohorts were used for external validation. One validation set included 109 melanoma samples from GSE91061 in the GEO database. The other included 153 samples from phs000452, which was obtained from the TIDE database. These two cohorts were used to examine the links between survival and immune-related features. Ensembl gene identifiers were mapped to the official gene symbols to ensure consistency across datasets. Genes expressed in <50% of the samples were excluded prior to downstream analyses. In addition, a single-cell transcriptomic dataset (GSE115978) was obtained from the GEO database to support cell-level analyses.

### 2.2 Consensus unsupervised clustering

Unsupervised clustering was performed based on TM-related expression profiles using the ConsensusClusterPlus package in R. Sample similarity was calculated using k-means clustering with Euclidean distance, and cluster stability was evaluated through 1,000 resampling iterations with 80% sample resampling. Cumulative distribution function curves together with consensus metrics were used to determine the most suitable number of clusters. Differences in clinical and molecular features among clusters were visualized with heatmaps generated using the pheatmap package. Principal component analysis was also performed to show sample distribution and the separation between clusters.

### 2.3 Analysis of somatic mutations in SKCM

Differences in TMRG expression between melanoma and normal skin tissues were tested using nonparametric methods. Mutation patterns in the identified subtypes were summarized and plotted with the maftools package in R. Tumor mutational burden (TMB) was also calculated for each sample to compare the overall mutation level among different subtypes.

### 2.4 Functional enrichment of TMRGs

STRING was used to obtain protein interaction information for TMRGs. Differential expression across the molecular subtypes was analyzed using linear modeling, and genes with |log2 fold change| > 1 and FDR < 0.05 were retained. Functional analyses were subsequently performed for these genes, including Gene Ontology analysis, Kyoto Encyclopedia of Genes and Genomes analysis, and gene set enrichment analysis, through clusterProfiler.

### 2.5 Evaluation of immune characteristics

The immune features of the tumor samples were assessed using several computational methods. Immune and stromal scores were estimated using the ESTIMATE method, while the proportions of different immune cell types were calculated using the CIBERSORT algorithm. Expression levels of immune checkpoint-related genes and human leukocyte antigen-associated genes were analyzed across molecular subtypes to identify potential differences relevant to immunotherapy. Hypoxia scores were obtained from the cBioPortal database.

### 2.6 Construction of a TMRG-based risk score

A prognostic risk model based on TMRGs was established. Genes related to OS were first screened using univariate Cox regression analysis with a threshold of p < 0.05. LASSO regression was then applied to reduce collinearity, and stepwise selection based on the Akaike information criterion (AIC) was used to identify the final gene set. The selected genes were entered into a multivariate Cox regression model, and the risk score for each patient was calculated as follows:


$$\text{Risk score}=\sum_{i=1}^{N}(Expi\times Coei)$$

Patients were divided into high- and low-risk groups according to the median risk score. Survival differences between the groups were compared using Kaplan–Meier analysis and the log-rank test. The model was further tested in an independent external cohort.

### 2.7 Clinical prognostic model development

Variables that remained significant in univariate and multivariate Cox regression analyses were used to build a nomogram for predicting 1-, 3-, and 5-year OS. Decision curve analysis (DCA) was performed to determine whether the nomogram provided clinical benefit over a range of threshold probabilities.

### 2.8 Single-cell transcriptomic analysis

Single-cell RNA sequencing data were analyzed with Seurat. Cells with mitochondrial gene content of >10% or <50 detected genes were excluded. The top 1,500 HVGs were selected for dimensionality reduction. Clustering was conducted using the first 15 PCs and cluster-specific markers were identified.

### 2.9 Immunotherapy-related signatures and drug response prediction

To investigate immunotherapy-related differences, TIDE scores were calculated for the high- and low-risk groups and used to reflect checkpoint-associated immune features. Potential drug response was estimated with OncoPredict on the basis of GDSC data, with IC50 values used as the indicator of sensitivity. Group comparisons were carried out using nonparametric tests.

### 2.10 Cell culture and transfection

SK-MEL-28 and A375 cells were grown in RPMI-1640 or DMEM containing 10% fetal bovine serum, penicillin (100 U/mL), and streptomycin (100 μg/mL). HaCaT cells were cultured in DMEM with the same additives. All cell lines were maintained at 37°C under 5% CO_2_.

For knockdown experiments, melanoma cells were transfected with siRNA targeting IDO1 using RNAiMAX (Thermo, USA) according to the supplier’s protocol. Subsequent functional tests were carried out 24 hours later. The siRNA sequences are provided in **Supplementary Table 1**.

### 2.11 Expression of mRNA and protein

Total RNA was isolated using RNAiso Plus (Takara, Japan). Reverse transcription was performed with the PrimeScript RT Reagent Kit (Takara) to obtain cDNA. qPCR was subsequently carried out with TB Green Premix Ex Taq II (Takara) as described previously.

For protein analysis, samples were separated using SDS-PAGE and examined using western blotting following standard methods. The signal intensity of each band was quantified with Image Lab 4.0 software (Bio-Rad, Austria). Protein expression was normalized against the corresponding loading control or total protein level. Details of the primers and antibodies are shown in **Supplementary Table 2**, **S3**.

### 2.12 Cell proliferation assay

For cell proliferation assay, cells in the logarithmic phase were seeded in 96-well plates at 3 × 10^3^ cells per well. Three parallel wells were prepared for each group and maintained at 37°C. At the indicated time points (0, 24, 48, 72, and 96 hours), CCK-8 reagent (Biosharp, China) was added, and the plates were incubated for another 1 hour. Blank wells containing only medium and CCK-8 reagent were included. Optical density was measured at 450 nm using a microplate reader.

### 2.13 Transwell assay

Cell migration and invasion were examined with Transwell chambers. A total of 5 × 10^4^ cells were added to each upper chamber, and the lower chamber contained medium supplemented with 10% serum. After 24 hours of incubation, the cells that passed through the membrane were fixed, stained with 0.1% crystal violet for 10 minutes, and photographed under identical conditions. For invasion testing, the membranes were first coated with Matrigel (Corning, USA), which had been diluted to 1 mg/mL in serum-free medium. The coated inserts were incubated at 37°C for 4 hours and then left at 4°C overnight before use.

### 2.14 Apoptosis assay

Apoptosis was analyzed using the Annexin V-FITC/PI apoptosis kit (Elabscience, China) following the manufacturer’s instructions. Flow cytometry was performed on a CytoFLEX instrument (Beckman Coulter), and the data were analyzed with FlowJo.

### 2.15 Metabolic characterization

For ROS detection, cells were cultured and treated as indicated and then incubated with a fluorescent ROS probe provided in a commercial kit (Beyotime, China). After washing off excess dye, fluorescence was examined immediately using microscopy or recorded with a microplate reader.

Mitochondrial membrane potential was examined using the JC-1 method with a commercial kit (Beyotime). After treatment, cells were stained with JC-1 working solution in the dark, washed, and observed under a fluorescence microscope. Membrane potential changes were reflected by the shift between red and green fluorescence.

Oxygen consumption rate (OCR) and ECAR were assessed with commercial fluorescence-based kits (Elabscience; Solarbio, China). Cells were seeded in 96-well plates, treated as required, and then incubated with the corresponding detection reagents. Fluorescence signals were measured using a microplate reader, and the results were corrected by cell number or protein concentration.

### 2.16 Enzymatic activity and metabolite quantification

Caspase-3 activity was tested with a colorimetric kit (Beyotime). Trp and kynurenine levels in cell samples were determined using ELISA kits from ELK Biotechnology and FineTest. The measured values were normalized to total protein level.

### 2.17 Statistical analysis

R was used for bioinformatic analysis and GraphPad Prism 10 (GraphPad, USA) was used for the analysis of experimental results. Differences between groups were evaluated with the Wilcoxon rank-sum test or unpaired Student’s *t*-test, as suitable. For analyses involving more than two groups, the Kruskal–Wallis test or one-way ANOVA was applied as needed. OS was analyzed with the Kaplan–Meier method and compared using the log-rank test. Each *in vitro* assay included at least three independent biological repeats. Results are expressed as mean ± SD, and results with two-sided p < 0.05 were considered statistically significant.

## 3 Results

### 3.1 Variation in TM gene expression among patients with SKCM

**Figure 1:**
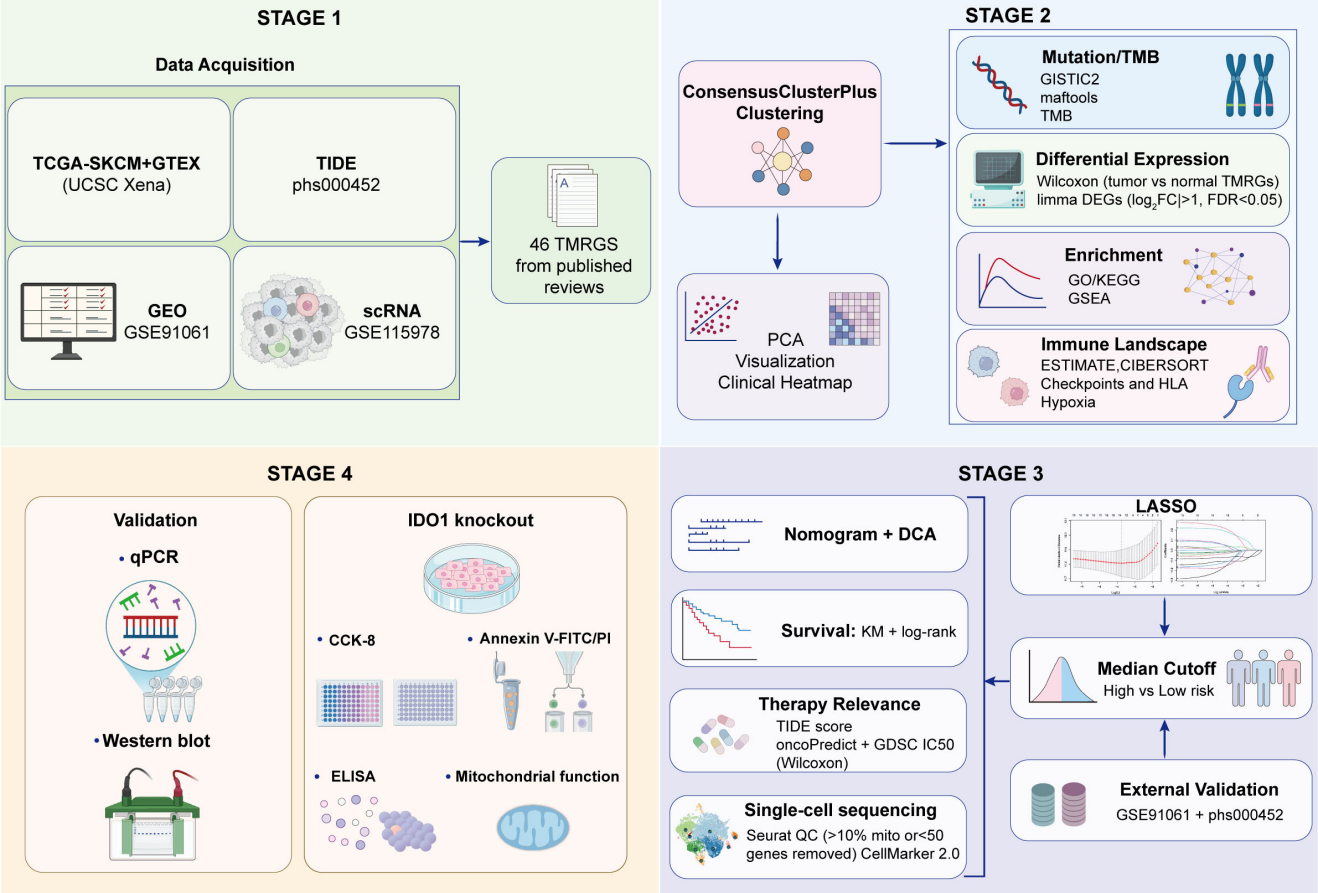
Flowchart of this study.

**Figure 2:**
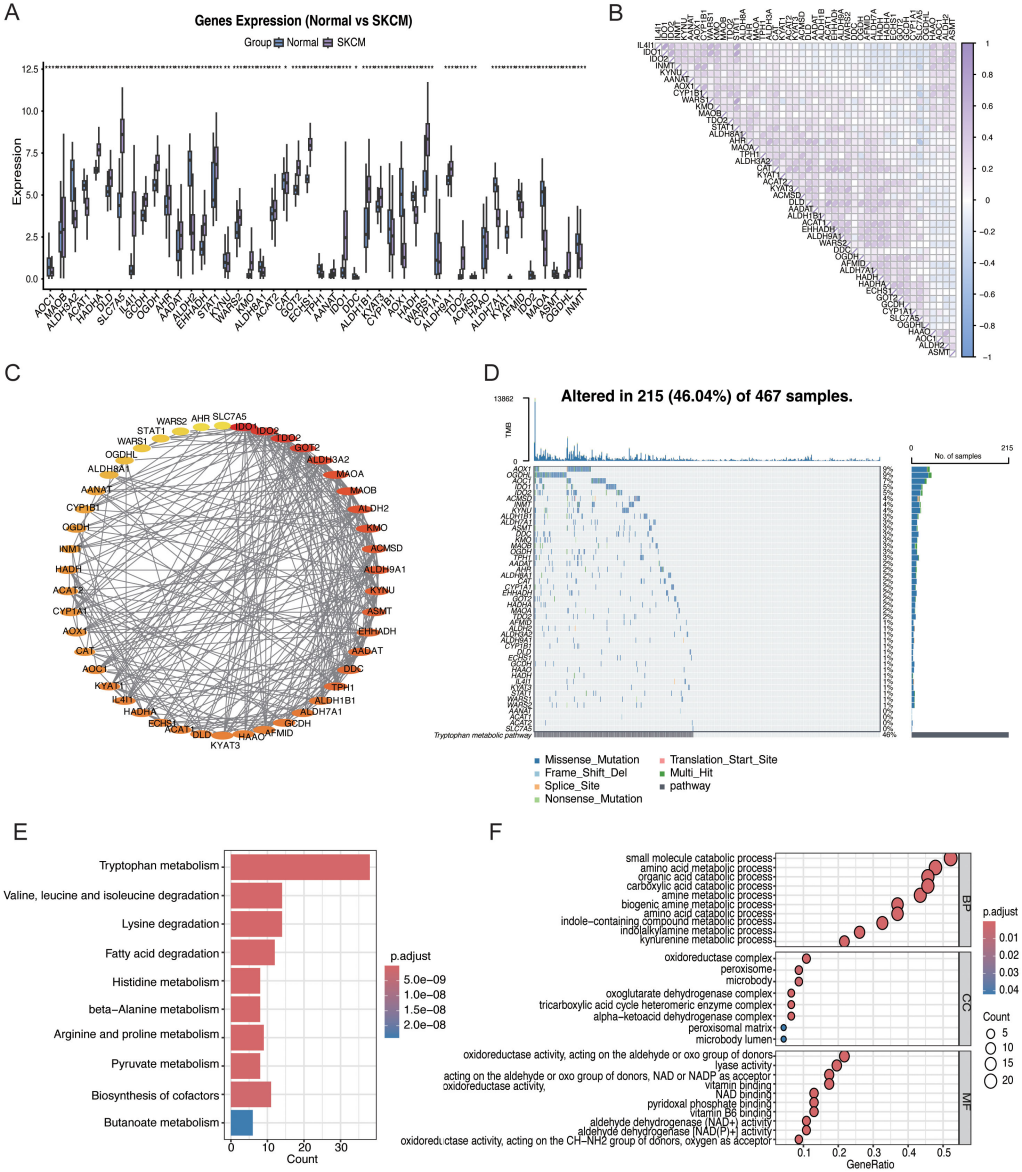
Landscape of TMRGs in skin cutaneous melanoma (SKCM). **(A)** TMRG expression in normal and SKCM tissues. **(B)** Correlation among the TMRGs in SKCM. **(C)** Interaction network of TMRGs. **(D)** TMRG mutation landscape in SKCM. **(E)** Gene ontology enrichment of TMRGs. **(F)** Kyoto Encyclopedia of Genes and Genomes pathways of the TMRGs. *Statistical significance is indicated as follows: *p < 0.05, **p < 0.01, ***p < 0.001.

**Figure 1** presents a flowchart of this study. Most TMRGs were expressed differently between SKCM and normal skin tissues (**Figure 2A**). The expression of several genes, including *AOC1*, *MAOB*, *ALDH3A2*, *ACAT1*, *HADHA*, and *DLD*, was markedly dysregulated in tumor tissues compared with that in normal skin tissue. The correlation analysis revealed strong associations among the TMRGs (**Figure 2B**). Notably, *IDO1* expression strongly positively correlated with *IDO2* and *STAT1* expression, whereas *INMT* expression showed a strong correlation with *AOX1* expression, suggesting coordinated regulation of genes involved in related metabolic pathways.

To better understand functional connections among these TMRGs, a PPI network was built using the TMRGs (**Figure 2C**). The network analysis identified IDO1, IDO2, TDO2, KMO, and KYNU as central hub genes, indicating their potential roles in metabolic regulation in SKCM. Analysis of somatic mutations showed that missense mutation was the most frequent alteration type in SKCM (**Figure 2D**). The five genes with the highest mutation frequencies were *AOX1*, *OGDHL*, *AOC1*, *IDO1*, and *IDO2*. The functional enrichment analysis further showed that these TMRGs are mainly linked to metabolism- and redox-related biological processes (**Figures 2E, F**), including Trp metabolism, oxidoreductase complex activity, pyruvate metabolism, amino acid catabolic processes, small-molecule metabolism, and histidine metabolism.

### 3.2 TMRG-based molecular classification of SKCM

**Figure 3:**
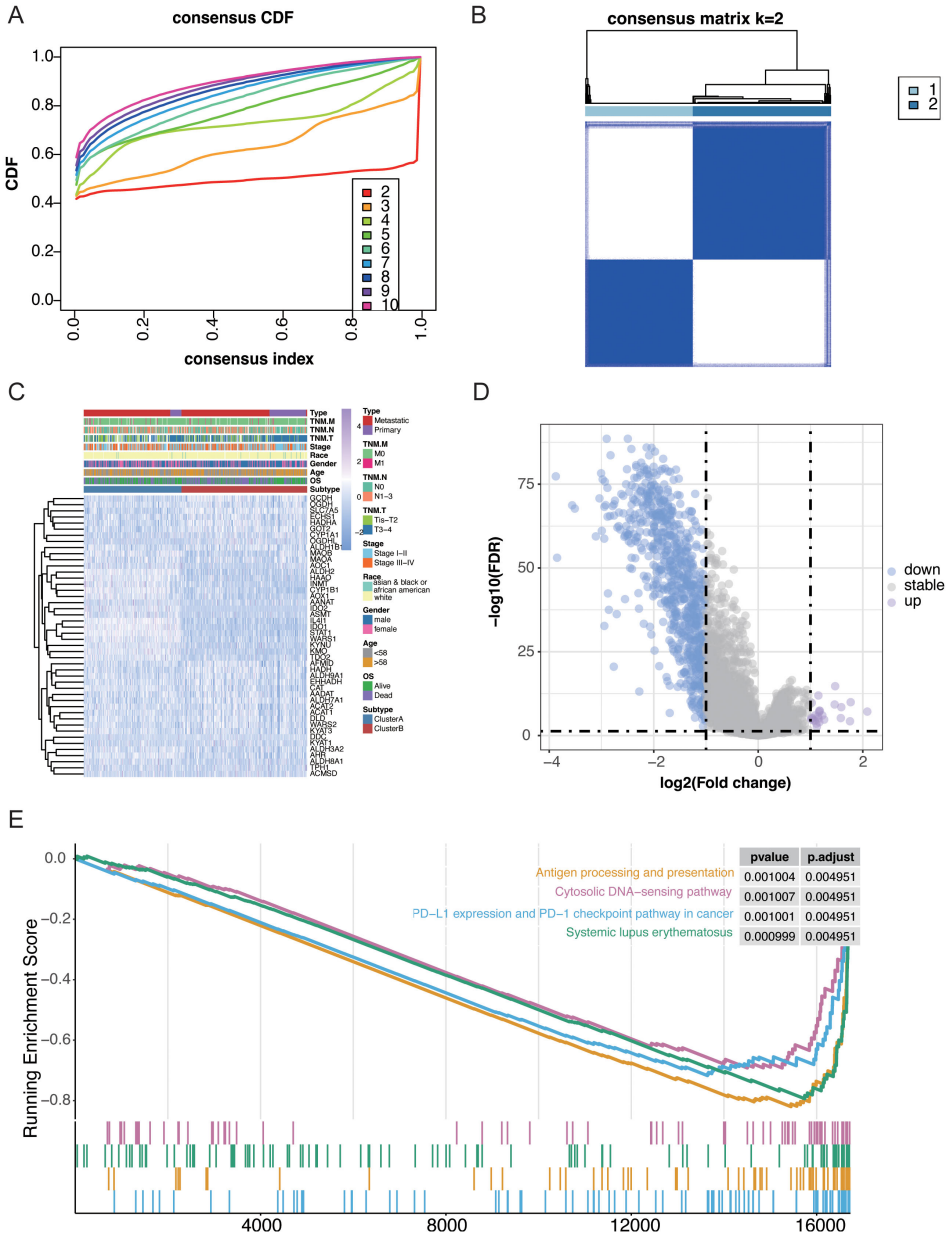
Consensus clustering and biological characterization of tryptophan metabolism-related gene (TMRG)-based subtypes in skin cutaneous melanoma (SKCM). **(A)** Cumulative distribution function curves for subtype determination. **(B)** Consensus matrix identifying two TMRG-based subtypes. **(C)** Clinical characteristics and gene expression of TMRG subtypes. **(D)** Differentially expressed genes between TMRG subtypes. **(E)** Gene set enrichment analysis of immune-related pathways between TMRG subtypes.

Patients with SKCM were classified on the basis of TMRG expression patterns using consensus clustering of a cohort of 457 cases. Evaluation of different clustering options supported k = 2 as the most stable grouping scheme. Accordingly, the samples were assigned to two subtypes, namely Cluster A (n = 200) and Cluster B (n = 257) (**Figures 3A, B**). Their clinicopathological features, including cancer type, TNM stage, overall stage, race, sex, age, and survival status, were then summarized and compared, as shown in the heatmap (**Figure 3C**). These analyses indicated that the two subtypes differed clearly in their clinical composition.

To further define the biological features of the two subtypes, GSEA was performed. The results showed that Cluster B was strongly linked to immune-associated functions, with significant enrichment in pathways related to antigen processing and presentation, PD-L1 expression, and PD-1 checkpoint signaling in cancer (**Figures 3D, E**). In contrast, Cluster A showed only a small number of differentially expressed genes, and no significantly enriched pathway was detected.

### 3.3 Analysis of the tumor immune microenvironment between TM-related subtypes

**Figure 4:**
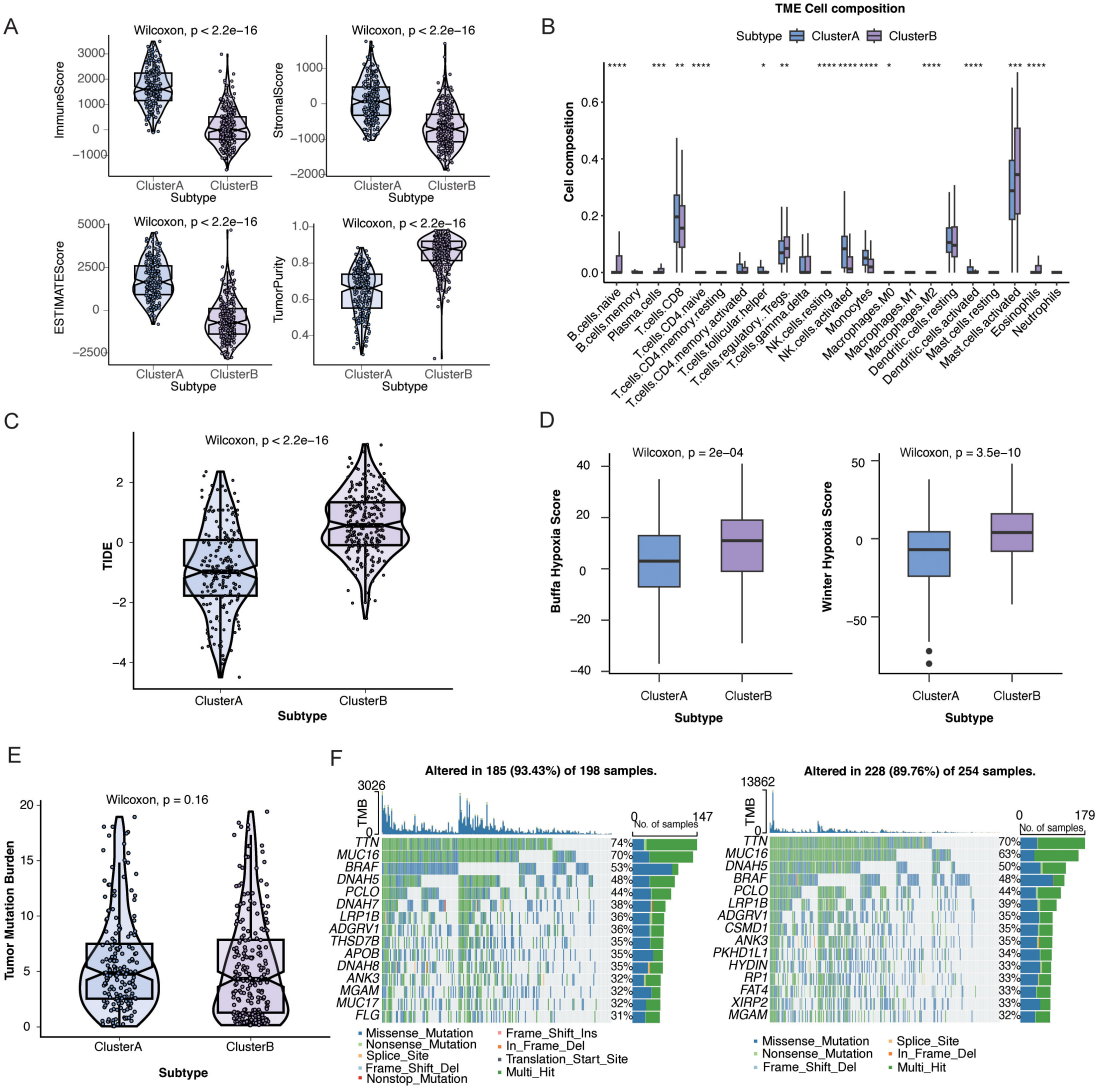
Immune, genomic, and therapeutic features of tryptophan metabolism-related gene (TMRG)-based subtypes in SKCM. **(A)** Tumor microenvironment cell composition among the TMRG subtypes. **(B)** ESTIMATE scores of the TMRG subtypes. **(C)** TIDE scores of the TMRG subtypes. **(D)** Hypoxia scores of the TMRG subtypes. **(E)** Tumor mutation burden of the TMRG subtypes. **(F)** Mutation landscape of Cluster A and Cluster B. *Statistical significance is indicated as follows: *p < 0.05, **p < 0.01, ***p < 0.001, ****p < 0.0001 .

To characterize immune landscape differences between the two TM-related subtypes, immune score, stromal score, and ESTIMATE score were compared. Cluster A displayed significantly higher immune, stromal, and ESTIMATE scores, whereas Cluster B showed higher tumor purity (**Figure 4A**). Cluster A was dominated by effector and antigen-presenting immune populations such as CD8+ T cells, activated NK cells, monocytes, and activated dendritic cells. In contrast, Cluster B demonstrated a relative enrichment of immunoregulatory and inflammation-associated cells, including plasma cells, regulatory T cells, activated mast cells, and eosinophils (**Figure 4B**). Cluster B exhibited significantly higher TIDE scores than Cluster A, indicating a more immune-evasive phenotype (**Figure 4C**), and also showed a stronger hypoxia signature (**Figure 4D**). TMB did not differ significantly between the subtypes (p = 0.16; **Figure 4E**). Moreover, the overall somatic mutation landscape was largely comparable across the clusters. The 20 most frequently mutated genes are shown in **Figure 4F**. Representative genes, including *AOX1*, *AOC1*, *IDO1*, *IDO2*, and *OGDHL*, had similar mutation frequencies in the two subtypes (**Supplementary Figure 1**).

### 3.4 Identification of core TMRGs and development of a prognostic signature

**Figure 5:**
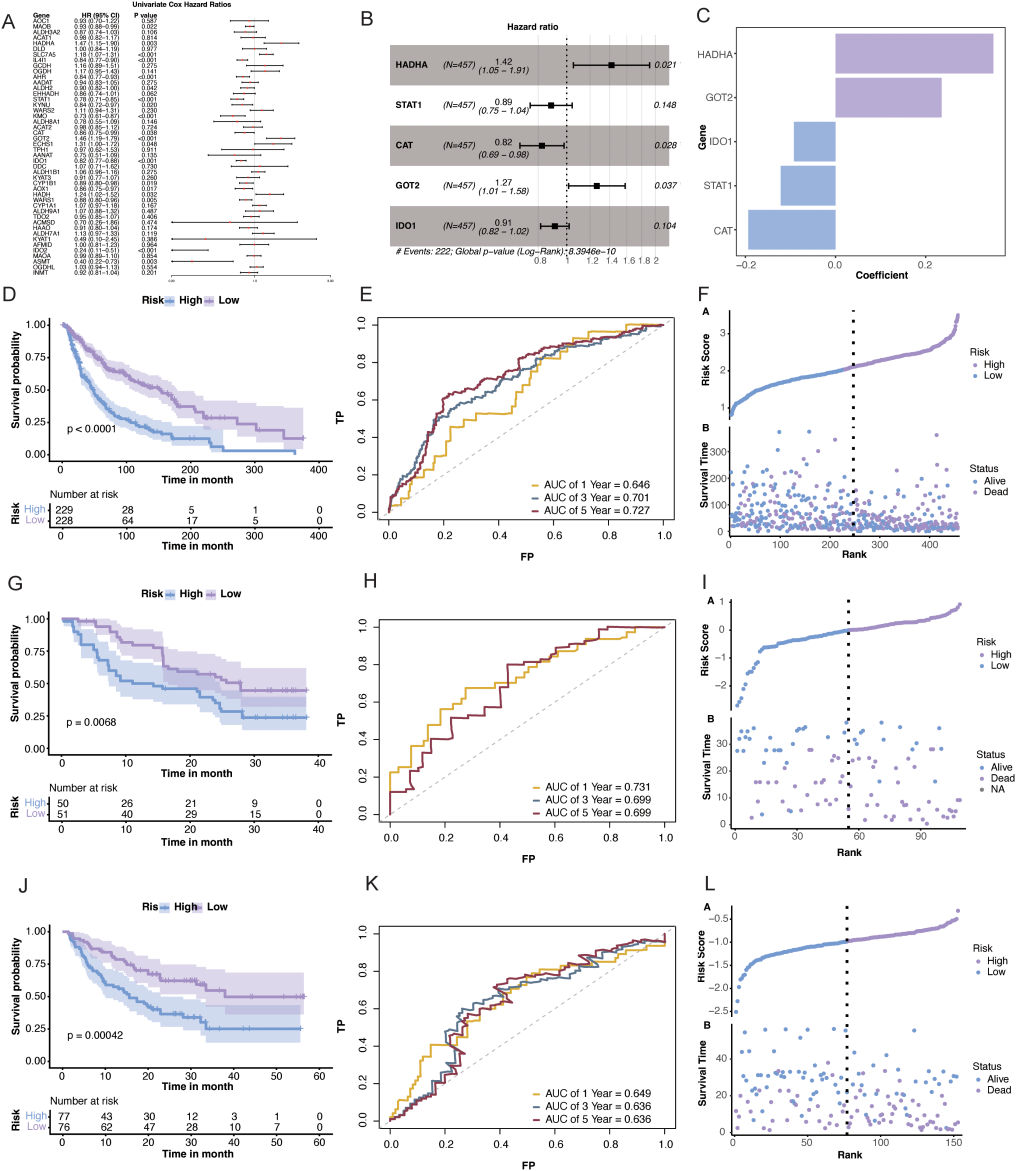
Prognostic value of the tryptophan metabolism-related gene (TMRG)-based risk model in skin cutaneous melanoma (SKCM). **(A)** Univariate Cox analysis of TMRGs. **(B)** Stepwise AIC regression analysis defining the final five prognostic genes for risk model construction. **(C)** Regression coefficients of TMRGs related to the prognosis of SKCM. **(D, G, J)** Kaplan–Meier analysis of overall survival in TCGA-SKCM, GSE91061, and phs000452 cohorts. **(E, H, K)** ROC curves of the risk model in TCGA-SKCM, GSE91061, and phs000452 cohorts. **(F, I, L)** Risk score distribution and survival status in TCGA-SKCM, GSE91061, and phs000452 cohorts.

The univariate Cox regression analysis of TMRGs in TCGA-SKCM database revealed 19 genes significantly associated with OS, and they were retained as candidate prognostic genes for subsequent model construction (**Figure 5A**). To reduce model complexity, LASSO Cox regression was subsequently applied, which reduced the candidate set from 19 to 12 genes (**Supplementary Figure 2**). These genes were further refined through stepwise selection based on the AIC, resulting in a five-gene signature comprising *HADHA*, *STAT1*, *CAT*, *GOT2*, and *IDO1* (**Figure 5B**). A multivariable Cox regression model incorporating the selected genes was used to derive an individual risk score. This score was computed as the sum of expression values multiplied by their respective regression coefficients: risk score = (0.3502 × HADHA) + (0.2355 × GOT2) − (0.1216 × STAT1) − (0.1938 × CAT) − (0.0924 × IDO1) (**Figure 5C**).

Patients were then split into high- and low-risk groups according to the median risk score. Kaplan–Meier analysis showed that the low-risk group had clearly better OS in TCGA-SKCM cohort (p < 0.0001; **Figure 5D**). The same pattern was observed in the GSE91061 (p = 0.0068) and phs000452 (p = 0.00042) cohorts (**Figures 5G, J**). The ROC analysis further supported the predictive ability of the signature. In TCGA-SKCM cohort, the AUC values at 1, 3, and 5 years were 0.646, 0.701, and 0.727, respectively (**Figure 5E**). The AUC values were 0.731, 0.699, and 0.699 in GSE91061 (**Figure 5H**), and 0.649, 0.636, and 0.636 in the phs000452 cohort (**Figure 5K**). The distributions of risk score and survival status in the three cohorts are shown in **Figures 5F, I, L**.

### 3.5 Development and performance of the nomogram

**Figure 6:**
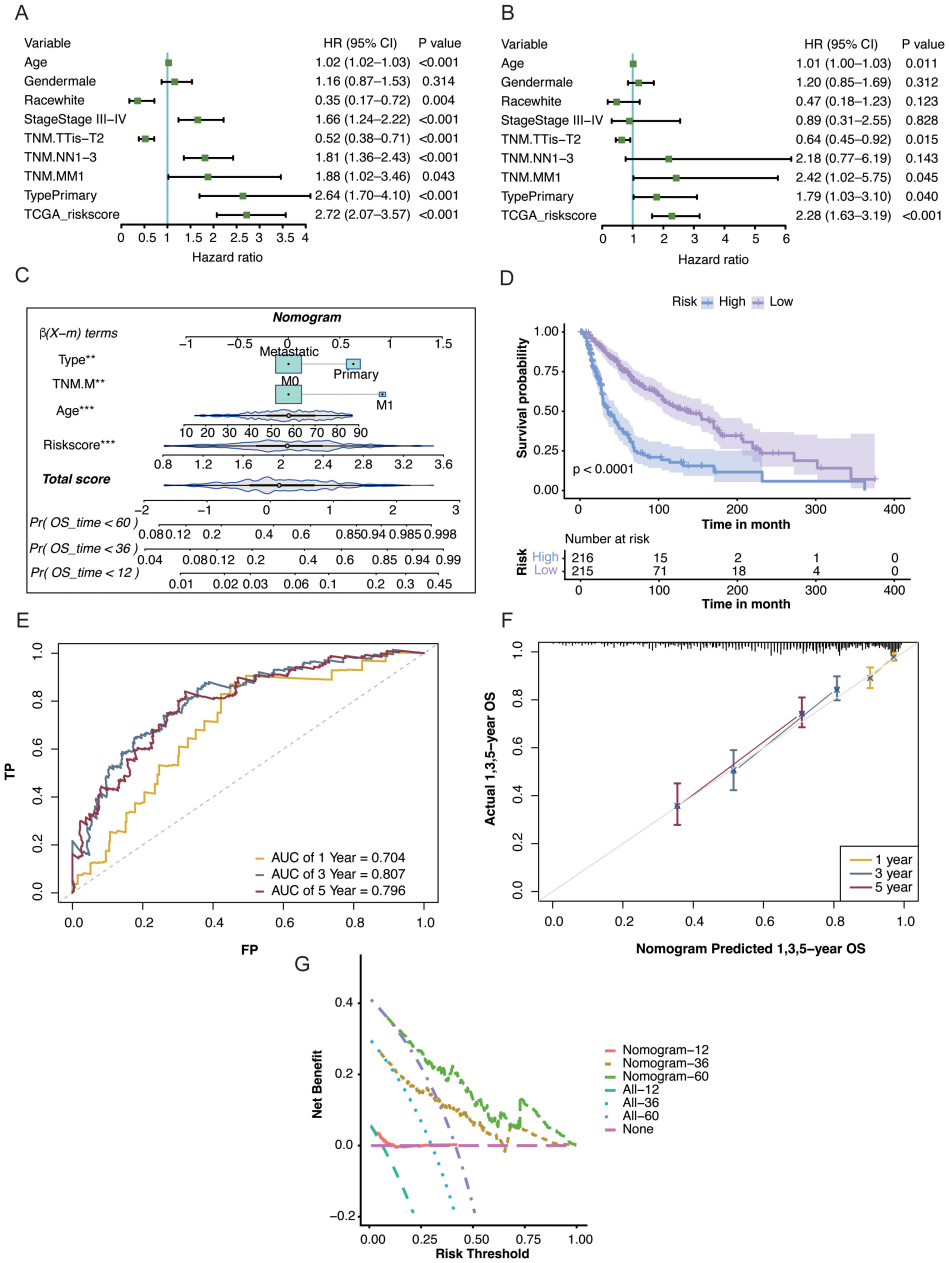
Nomogram construction and performance assessment based on the tryptophan metabolism-related gene (TMRG) risk score. **(A)** Univariable Cox analysis of clinical variables and the TMRG risk score for overall survival **(OS)** in TCGA-SKCM cohort. **(B)** Multivariable Cox analysis identifying the independent prognostic factors. **(C)** Nomogram combining the TMRG risk score and clinical variables to estimate OS. **(D)** KM curves stratified by the nomogram-defined risk groups. **(E)** Receiver operating characteristic (ROC) curves at 1, 3, and 5 years. **(F)** Calibration for predicted versus observed OS. **(G)** Decision curve analysis (DCA) comparing the net benefit of the nomogram.

To test whether the TMRG-derived risk score could improve survival prediction when combined with clinical factors, we performed Cox regression analyses in TCGA-SKCM cohort. Age, TNM stage, cancer type, and the risk score were significantly associated with OS (**Figures 6A, B**). A nomogram was then established using these variables to estimate the probability of 1-, 3-, and 5-year survival in patients with SKCM (**Figure 6C**).

Survival curves based on the nomogram showed a clear separation between the groups, and patients in the high-risk group had significantly worse survival than those in the low-risk group (p < 0.001; **Figure 6D**). The predictive performance of the nomogram was further assessed using time-dependent ROC analysis, which yielded AUC values of 0.704, 0.807, and 0.796 at 1, 3, and 5 years, respectively (**Figure 6E**). Calibration plots showed that the predicted survival probabilities were close to the observed outcomes at each time point (**Figure 6F**). Decision curve analysis further indicated that the nomogram offered more net benefit than any single clinical factor across a broad range of threshold probabilities (**Figure 6G**).

### 3.6 Association between the TM-related prognostic model and the immune microenvironment

Immune infiltration patterns related to the TMRG-based prognostic model were explored in TCGA-SKCM cohort with CIBERSORT (**Supplementary Figure 3A**). Marked differences were observed between the high- and low-risk groups. The high-risk group had higher proportions of naïve B cells, Treg cells, resting NK cells, activated mast cells, and eosinophils, whereas the low-risk group had higher proportions of CD8+ T cells, follicular helper T cells, activated NK cells, monocytes, and activated dendritic cells. Associations between the five signature genes and immune infiltration were also evident, especially for NK-cell subsets (**Supplementary Figure 3B**). Activated NK cells showed negative correlations with HADHA and GOT2 expression, but positive correlations with STAT1, IDO1, and CAT expression; resting NK cells showed the opposite direction. In addition, CCL2, CCL17, and CD40 were more highly expressed in the low-risk group (**Supplementary Figures 3C–F**), further supporting a stronger immune-active state in this group.

**Figure 7:**
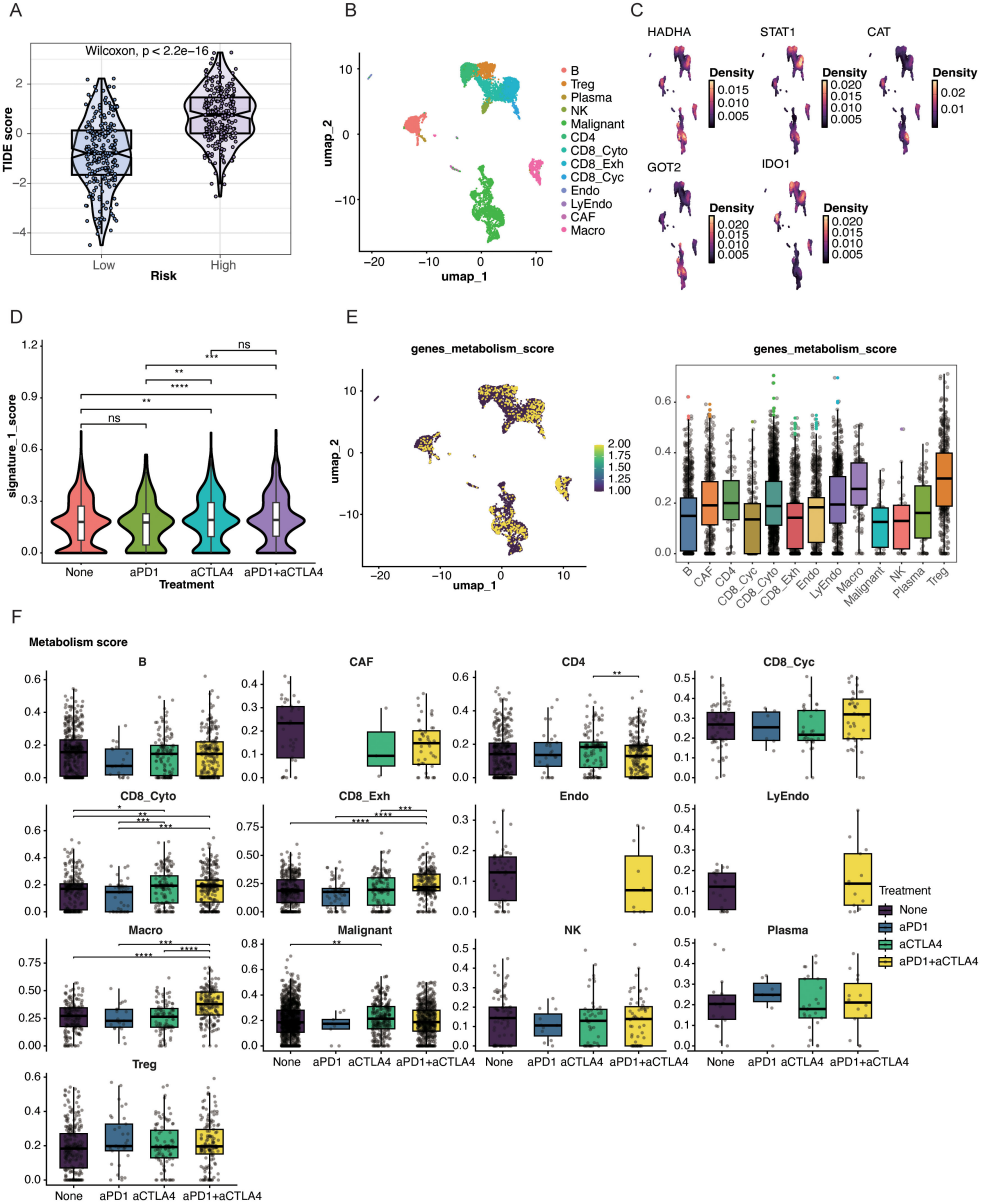
Single-cell characterization and immunotherapy relevance of the TMRGs. **(A)** TIDE scores for the high- and low-risk groups. **(B)** UMAP visualization of the annotated cell types in the single-cell RNA sequencing data. **(C)** The single-cell expression distribution of the five prognostic TMRGs. **(D)** Differences in the TMRG-related signature score across immunotherapy groups. **(E)** Comparison of TMRG metabolism scores across different cell types. **(F)** Cell type-specific changes in metabolism scores under different immunotherapy regimens. *Statistical significance is indicated as follows: *p < 0.05, **p < 0.01, ***p < 0.001, ****p < 0.0001; ns, no statistical significance (P > 0.05)..

The high-risk group had significantly higher TIDE scores, suggesting a stronger immune escape pattern and a possibly weaker response to immune checkpoint blockade (**Figure 7A**). To examine these findings at single-cell resolution, the GSE115978 cohort was further analyzed. Cell annotation and clustering results are shown in **Supplementary Figure 4**. The UMAP analysis showed that most genes in the prognostic model were mainly expressed in malignant cells and exhausted CD8+ T cells (**Figures 7B, C**).

We then compared the five-gene signature score across treatment groups in GSE115978 (**Figure 7D**). The untreated group and anti-PD-1 group showed similarly low signature gene activity, whereas the anti-CTLA-4 group and combined anti-PD-1 plus anti-CTLA-4 group showed higher activity. At the single-cell level, the five-gene signature had higher scores in Treg cells and macrophages, but lower scores in cytotoxic CD8+ T cells and NK cells (**Figure 7E**). We also examined the signature score across different treatment settings (**Figure 7F**). Compared with untreated patients, those receiving combined anti-PD-1 and anti-CTLA-4 therapy had significantly lower signature scores (p < 0.001). These results show the distribution of the five-gene signature across treatment groups, cell types, and therapeutic settings.

### 3.7 Drug sensitivity prediction

Drug response was predicted by comparing estimated IC50 values between the risk groups (**Supplementary Figure 5**). Several chemotherapeutic and targeted agents showed different response profiles. Notably, the high-risk group showed lower estimated IC50 values for cisplatin, axitinib, dabrafenib, epirubicin, gemcitabine, nilotinib, 5-fluorouracil, paclitaxel, and temozolomide, which may indicate increased drug sensitivity. These results suggest that the TMRG-based risk score is associated with variation in predicted therapeutic response.

### 3.8 Expression validation and selection of IDO1 for functional studies

**Figure 8:**
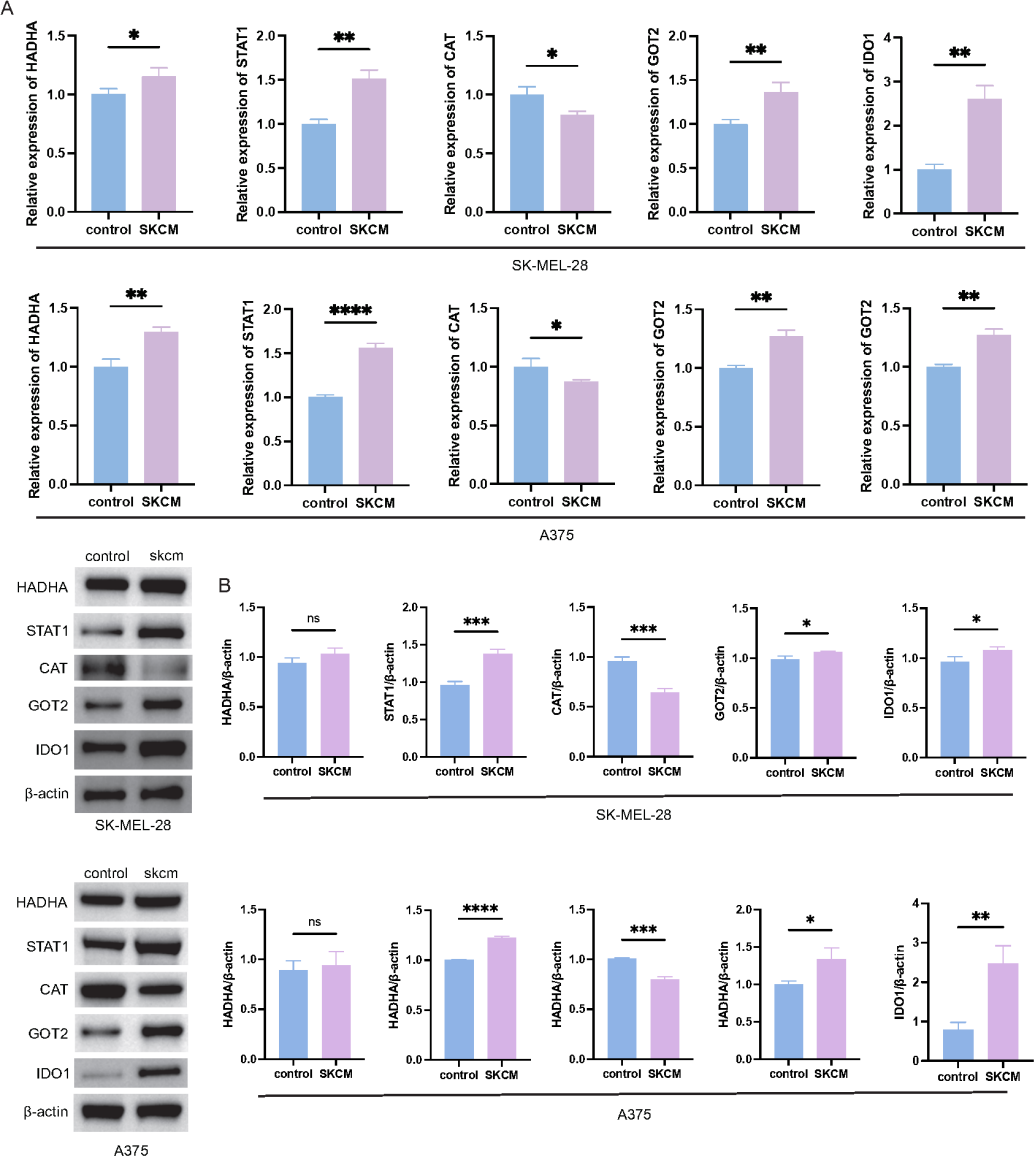
Validation of prognostic TMRG expression in melanoma cell lines. **(A)** qRT-PCR analysis of *HADHA*, *STAT1*, *CAT*, *GOT2*, and *IDO1* expression in SK-MEL-28 and A375 cells compared with that in the controls. **(B)** Western blot analysis and the corresponding quantification of HADHA, STAT1, CAT, GOT2, and IDO1 protein levels in SK-MEL-28 and A375 cells, with β-actin as the loading control. *Statistical significance is indicated as follows: *p < 0.05, **p < 0.01, ***p < 0.001, ****p < 0.0001; ns, no statistical significance (P > 0.05)..

The expression levels of the five TM-related prognostic genes (*HADHA*, *STAT1*, *CAT*, *GOT2*, and *IDO1*) were examined in the A375 and SK-MEL-28 melanoma cell lines, with HaCaT cells used as normal controls. The expression trends in melanoma cell lines were in line with those observed in TCGA-SKCM dataset (**Figure 8**). IDO1 was selected for further analysis because it plays a key rate-limiting role in the kynurenine pathway and is a hub gene in the TMRG-based PPI network. These characteristics make IDO1 a tractable candidate for functional follow-up, given its central role in the metabolism of Trp to kynurenine and its measurable downstream metabolites. Three independent siRNAs targeting IDO1 (siIDO1-1, siIDO1-2, and siIDO1-3) were transfected into melanoma cells. Their knockdown efficiency was evaluated; siIDO1–2 and siIDO1–3 showed more effective suppression of IDO1 expression. Therefore, these two siRNAs were used in the subsequent experiments (**Supplementary Figure 6**).

### 3.9 Effect of IDO1 silencing on melanoma cell behavior

**Figure 9:**
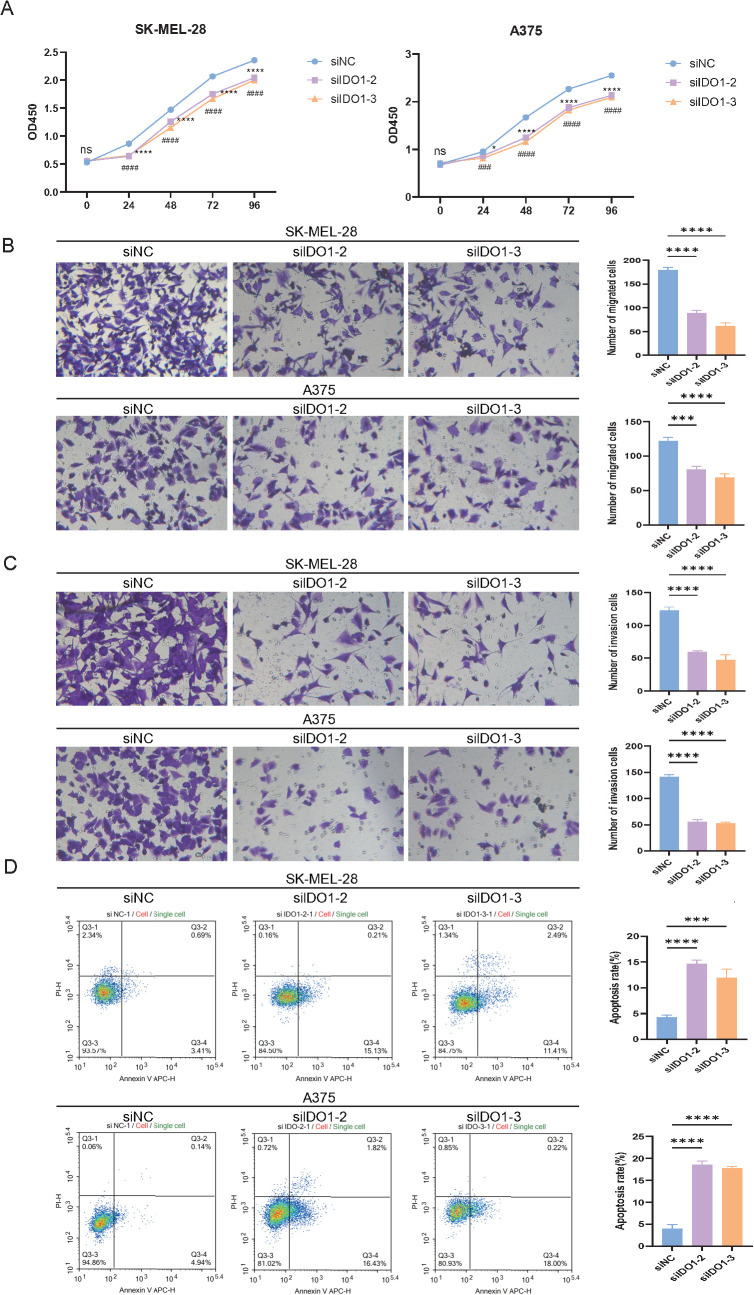
Effects of IDO1 knockdown on melanoma cell proliferation, motility, invasion, and apoptosis **(A)** Cell viability assessed using the CCK-8 assay in SK-MEL-28 and A375 cells after *IDO1* knockdown. **(B)** Transwell migration assay evaluating the migratory capacity of melanoma cells following *IDO1* silencing. **(C)** Transwell invasion assay assessing the invasive ability of the cells after *IDO1* knockdown. **(D)** Apoptosis analysis of melanoma cells via flow cytometry after *IDO1* silencing. *p < 0.05, ***p < 0.001, ****p < 0.0001. # denotes the statistical significance of the siIDO1-3 group compared to the siNC group (^###^P < 0.001, ^####^P < 0.0001.

The effect of *IDO1* depletion on melanoma cell behavior was evaluated by measuring their cell viability. The CCK-8 assay demonstrated a marked decrease in the viability of both A375 and SK-MEL-28 cells following *IDO1* knockdown compared with that of the siNC-treated control (p < 0.0001; **Figure 9A**). The Transwell assays showed that IDO1 knockdown markedly impaired both migration and invasion in A375 and SK-MEL-28 cells, with a significant reduction in migratory capacity (p < 0.001) and invasive capacity (p < 0.0001) (**Figures 9B, C**). Apoptotic responses were quantified using flow cytometry. IDO1-silenced cells displayed a substantially higher proportion of apoptotic events than control cells, indicating enhanced apoptosis upon *IDO1* inhibition (p < 0.0001; **Figure 9D**).

### 3.10 IDO1 knockdown is accompanied by alterations in mitochondrial function and cellular metabolism

**Figure 10:**
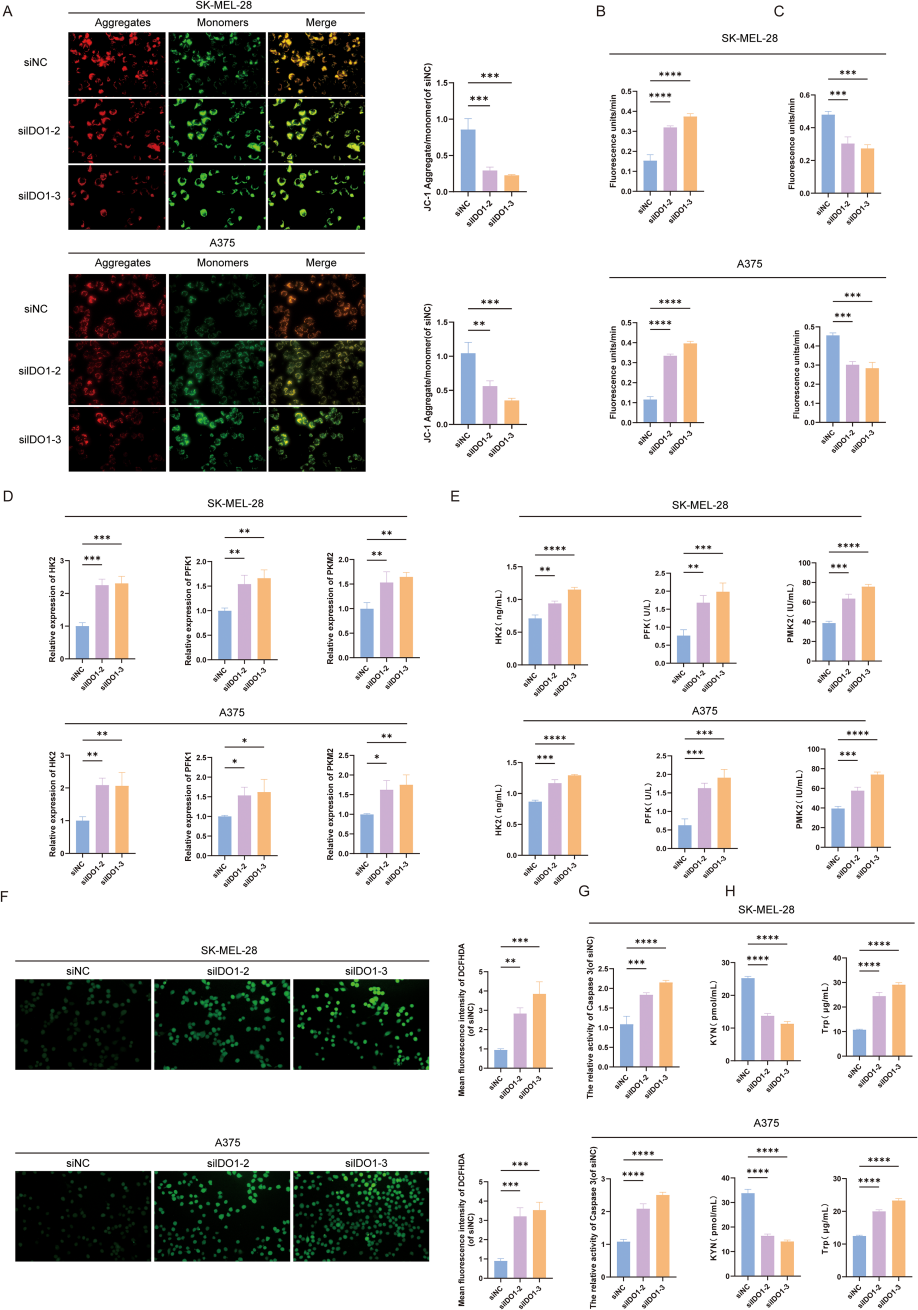
Effects of IDO1 knockdown on melanoma cell function and metabolism. **(A)** Analysis of mitochondrial membrane potential in siIDO1-treated cells as measured using JC-1 staining. **(B)** Extracellular acidification rate analysis following *IDO1* knockdown. **(C)** Oxygen consumption rate (OCR) analysis in melanoma cells after *IDO1* silencing. **(D)** Expression of genes related to glycolysis. **(E)** Changes in the activity of enzymes related to glycolysis. **(F)** Intracellular ROS levels detected via DCFH-DA fluorescence after *IDO1* knockdown. **(G)** Caspase-3 activity measured in melanoma cells transfected with siIDO1. **(H)** Intracellular kynurenine and tryptophan levels measured using ELISA following *IDO1* silencing. *Statistical significance is indicated as: *p < 0.05, **p < 0.01, ***p < 0.001, and ****p < 0.0001.

The observed loss of IDO1 expression was accompanied by marked changes in mitochondrial and metabolic features. The JC-1 assay showed a marked loss of mitochondrial membrane potential in IDO1-silenced cells, as indicated by a significantly reduced red/green fluorescence ratio (p < 0.0001; **Figure 10A**). At the metabolic level, cells lacking *IDO1* displayed a reprogrammed bioenergetic state with increased ECAR (p < 0.0001) and reduced OCR (p < 0.01; **Figures 10B, C**). Consistent with this metabolic shift, both the expression and enzymatic activities of key glycolytic enzymes, including HK2, PFK1, and PKM2, significantly increased after *IDO1* knockdown (**Figures 10D, E**). In line with these alterations, intracellular oxidative stress and executioner caspase activity were elevated, as determined by increased ROS levels (p < 0.01) and caspase-3 activity relative to those in the control cells (p < 0.0001; **Figures 10F, G**).

### 3.11 Alterations in Trp metabolism following IDO1 silencing

ELISA was used to determine intracellular Trp and kynurenine levels as indicators of Trp metabolism. Following IDO1 silencing, kynurenine level was significantly reduced, whereas intracellular Trp level was significantly increased relative to those in the control group (p < 0.01 and p < 0.0001, respectively; **Figure 10H**).

## 4 Discussion

We examined the TMRGs in SKCM by integrating large-scale transcriptomic data with clinical outcomes. By linking the gene expression profiles with survival data, we derived a TMRG-based risk score that effectively separated patients into groups with distinct prognostic trajectories. Differences in TMRG expression between melanoma and normal skin were evident and were preserved at the single-cell level, indicating that these transcriptional alterations persist across cellular contexts. Collectively, these findings suggest that the dysregulation of Trp metabolism reflects a coordinated metabolic state associated with molecular diversity and clinical heterogeneity in SKCM, rather than the effects of isolated prognostic markers.

A stepwise modeling method identified five prognostic genes—*HADHA*, *GOT2*, *STAT1*, *CAT*, and *IDO1*—which were used to construct a TMRG-based risk model. The risk score remained an independent predictor of OS after adjustment for standard clinical variables. When combined with clinical factors, this score helped estimate survival probability at multiple time points. These findings suggest that TM-related metabolic features may provide additional prognostic information beyond conventional clinicopathological indicators. The risk score was also associated with predicted responses to several chemotherapy and targeted drugs, suggesting that TM-based classification may be useful for treatment stratification.

Given the known immune regulatory role of Trp metabolism, particularly through the kynurenine pathway, we further investigated how the TMRG-based risk score was related to the tumor immune microenvironment. Tumors in the high-risk group were enriched in regulatory T cells and other immunosuppressive cell types, while low-risk tumors contained higher proportions of CD8+ T cells, activated NK cells, and dendritic cells with immune effector functions. Immune-related gene expression also showed clear differences between the groups. In addition, the higher TIDE scores in the high-risk group suggested a more immune-evasive state and a possibly weaker response to immune checkpoint therapy. Overall, these findings indicate that disordered Trp metabolism is closely associated with immune heterogeneity in SKCM.

The five-gene signature developed in our study may reflect an integrated metabolic–immune program in SKCM rather than the effects of the five marker genes. In our analysis, HADHA and GOT2 appeared to capture complementary aspects of mitochondrial metabolic adaptation. HADHA is a core enzyme involved in fatty acid β-oxidation, and it may reflect lipid utilization together with mitochondrial substrate handling; whereas, GOT2 is more closely associated with glutamine metabolism, aspartate production, and redox balance (19–22). Previous studies on melanoma have shown that high GOT2 expression is associated with worse prognosis; lower immune infiltration; lower immune, stromal, and ESTIMATE scores; and higher metabolic activity in tumor cells (23). Single-cell analyses have further indicated that GOT2 is predominantly expressed in malignant and stromal cells, and that *GOT2* silencing inhibits melanoma cell proliferation and colony formation (23). Taken together with the findings of prior research, the inclusion of *GOT2* and *HADHA* suggests that the model captures a broader mitochondrial metabolic axis spanning amino acid utilization and fatty acid oxidation (24, 25). In addition, *CAT* may represent the redox homeostasis component of this signature. Earlier reports have shown that CAT is strongly expressed in normal skin, especially in the epidermis, and participates in antioxidant defense and ultraviolet-related stress responses in melanocytes (26–29). Public melanoma dataset analyses have also suggested that CAT expression tends to decrease in primary lesions, implying that disruption of redox balance may accompany melanoma progression (30). In our model, the inclusion of CAT supports the possibility that oxidative stress adaptation is linked to the prognostic heterogeneity of SKCM, although this interpretation remains speculative and requires further validation.

STAT1 and IDO1 may represent the inflammatory and Trp-metabolic arm of the signature. Prior studies have shown that IFN-γ/STAT1 signaling can induce IDO1 expression, enhance Trp degradation and kynurenine production, and thereby suppress effector T-cell function while promoting an immunosuppressive microenvironment (31, 32). Studies have further suggested that IDO1 induction is shaped by not only inflammatory signaling but also crosstalk with oncogenic pathways (33). These findings are consistent with our observation that the prognostic value of the signature may derive from its ability to capture a broader metabolic–immune state associated with SKCM progression, rather than from the isolated contribution of any single gene.

This study had certain limitations that warrant consideration. First, the bioinformatic analyses were primarily conducted using retrospective public datasets, which may be affected by batch effects, sampling variability, and inter-cohort heterogeneity. Second, although IDO1 was selected for functional validation because it represents the most direct enzymatic link between the prognostic signature and the kynurenine branch of Trp metabolism, the remaining signature genes were not experimentally investigated in comparable depth. Therefore, the biological functions and potential interactions of the complete five-gene signature were not comprehensively validated in the present study. Third, the drug sensitivity analyses were based entirely on computational predictions rather than direct pharmacological testing, and therefore, the results require further validation before clinical translation. In addition, our experimental work was limited to two melanoma cell lines, and no animal or patient-derived xenograft studies were performed, which constrains the strength of causal inference and the overall robustness of the conclusions. Accordingly, future studies should include prospective clinical cohorts, expanded validation in additional melanoma models, direct testing of selected compounds, and *in vivo* studies such as xenograft or patient-derived xenograft models to strengthen mechanistic and translational relevance.

In summary, this study represents an integrative analysis linking Trp metabolism to the prognosis, immune microenvironmental features, and therapeutic sensitivity of SKCM. The TMRG-based risk score developed here highlights metabolic heterogeneity with potential clinical relevance, and offers a framework for further investigation of metabolism-informed risk stratification and treatment strategies for melanoma.

## Data Availability

The original experimental data supporting the conclusions of this article, including raw images of Western Blot, qPCR results, and cell functional assays, are deposited in the Figshare repository (DOI: 10.6084/m9.figshare.32230323). This study also analyzed publicly available datasets from TCGA (TCGA-SKCM), GEO (GSE91061 and GSE115978), and TIDE (phs000452). All data used in this research were obtained from these databases.
