# Supplementary material for: A tryptophan metabolism-related gene signature predicts prognosis and immune features in cutaneous melanoma
Source: Front Immunol. 2026 May 19;17:1806319. doi: 10.3389/fimmu.2026.1806319 (PMC13226596; doi:10.3389/fimmu.2026.1806319)
Supplement: Supplementary file 1 [file SupplementaryFile1.docx]

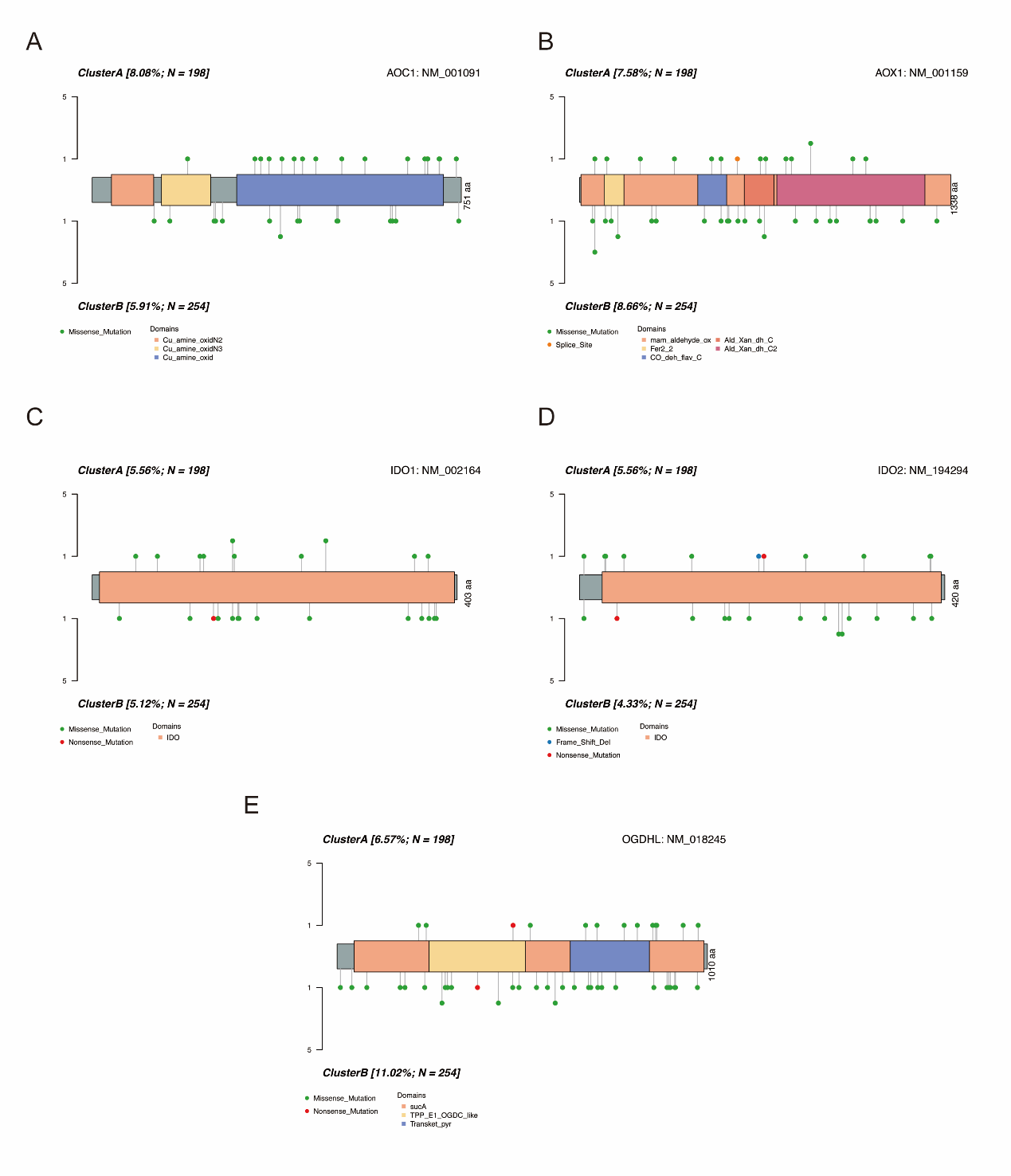


Figure 1. Somatic mutation landscapes of AOC1, AOX1, IDO1, IDO2, and OGDHL in Cluster A and Cluster B (TCGA-SKCM). Mutation types and protein domains are shown.


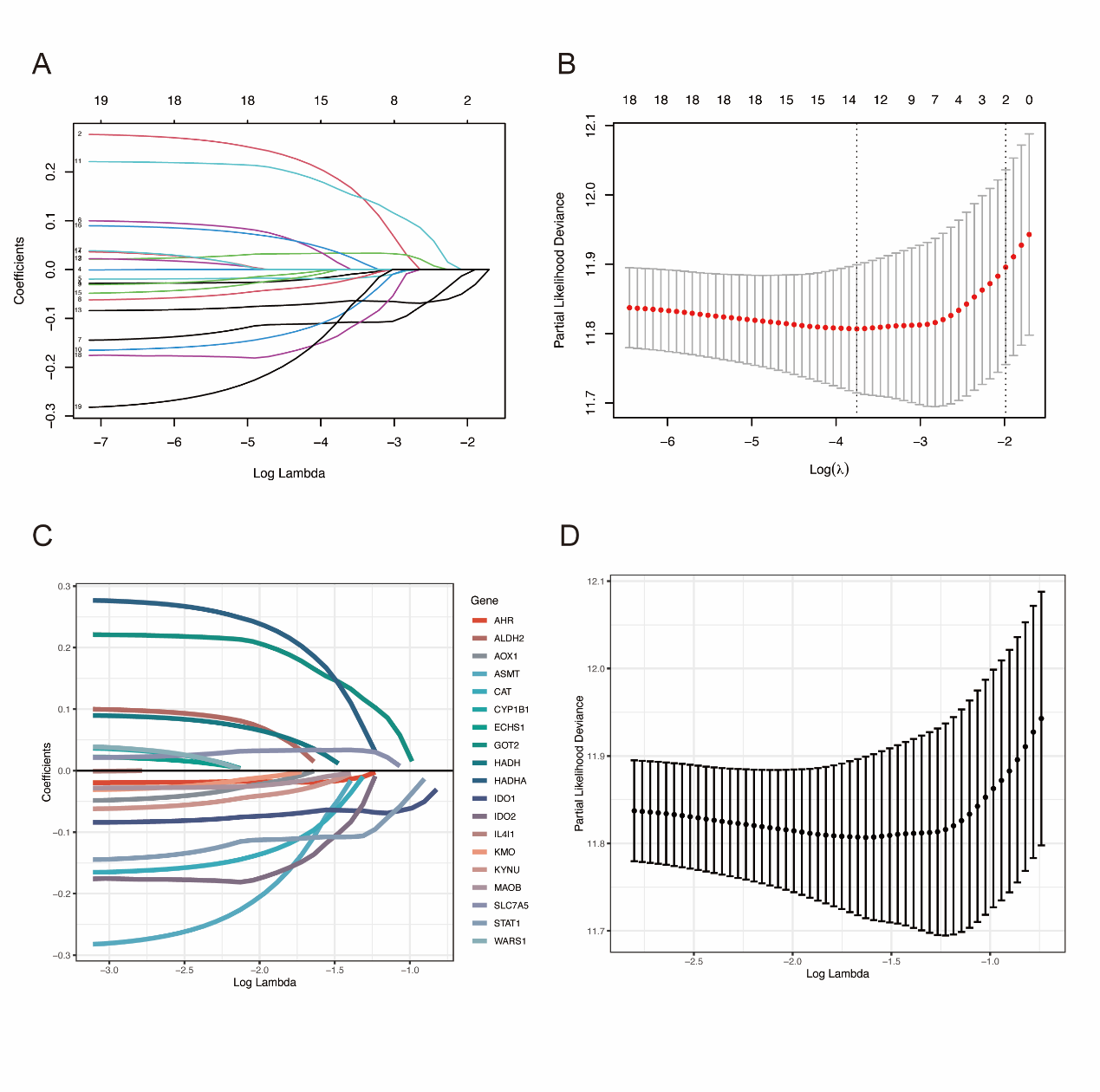


Figure 2. LASSO Cox regression for TMRG selection in TCGA-SKCM. (A) Coefficient paths. (B) Cross-validation for λ selection. (C) Gene coefficient profiles near optimal λ. (D) Partial likelihood deviance versus log(λ).


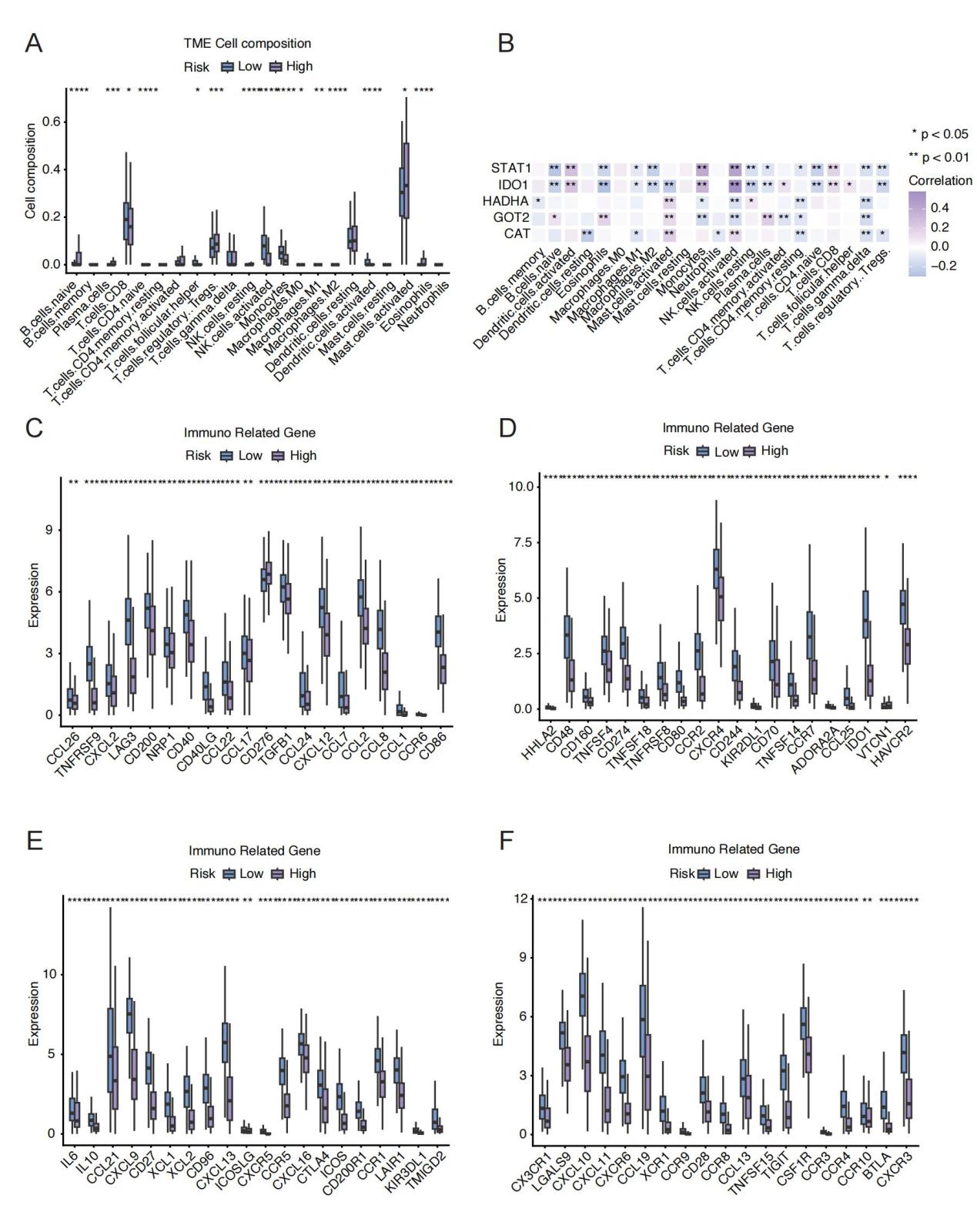


Figure 3. Immune features associated with the TM-related risk score in TCGA-SKCM. (A) Immune cell composition by CIBERSORT. (B) Correlations between signature genes and immune cells. (C–F) Differential expression of immune-related genes between risk groups.


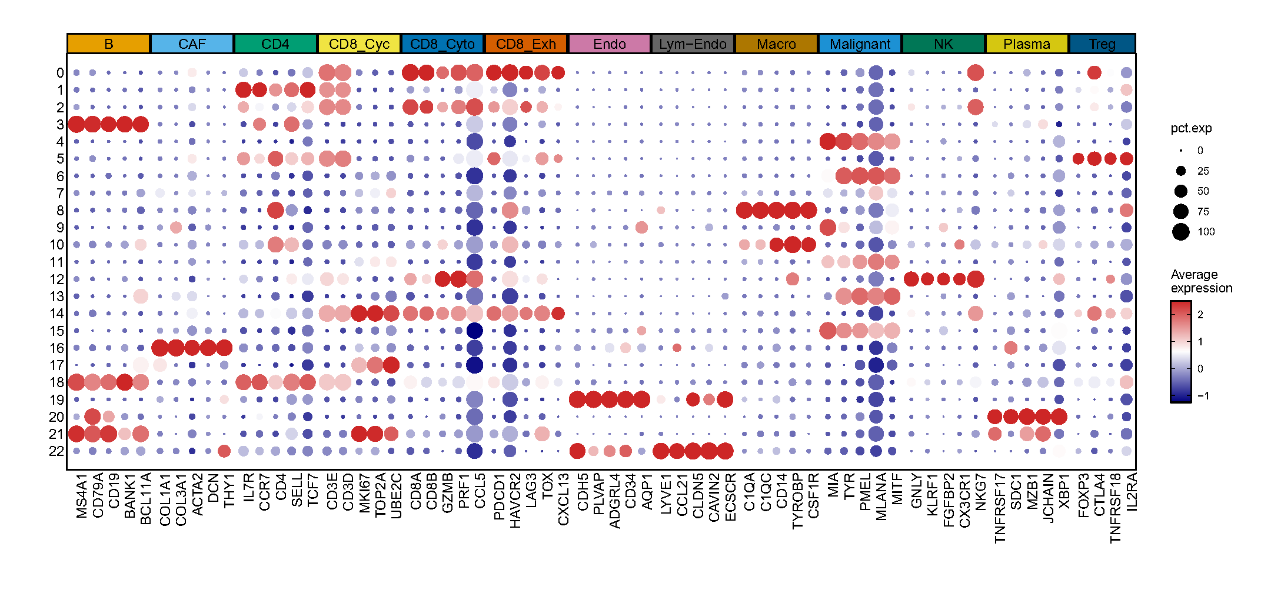


Figure 4. Dot plot showing marker gene expression across cell types.


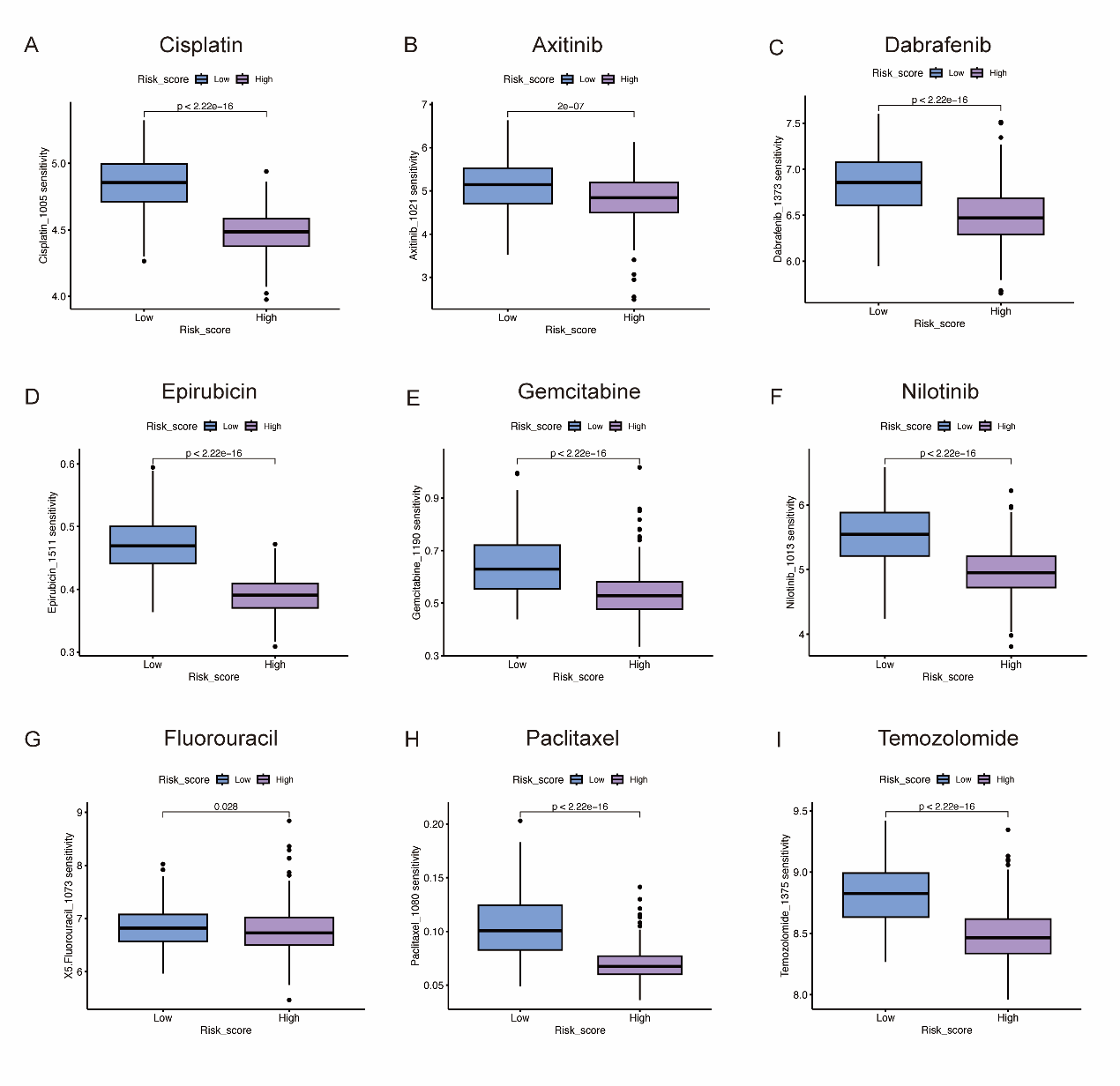


Figure 5. Predicted IC₅₀ differences between low- and high-risk groups for multiple agents in TCGA-SKCM. (A–I) Cisplatin, Axitinib, Dabrafenib, Epirubicin, Gemcitabine, Nilotinib, 5-Fluorouracil, Paclitaxel, and Temozolomide.


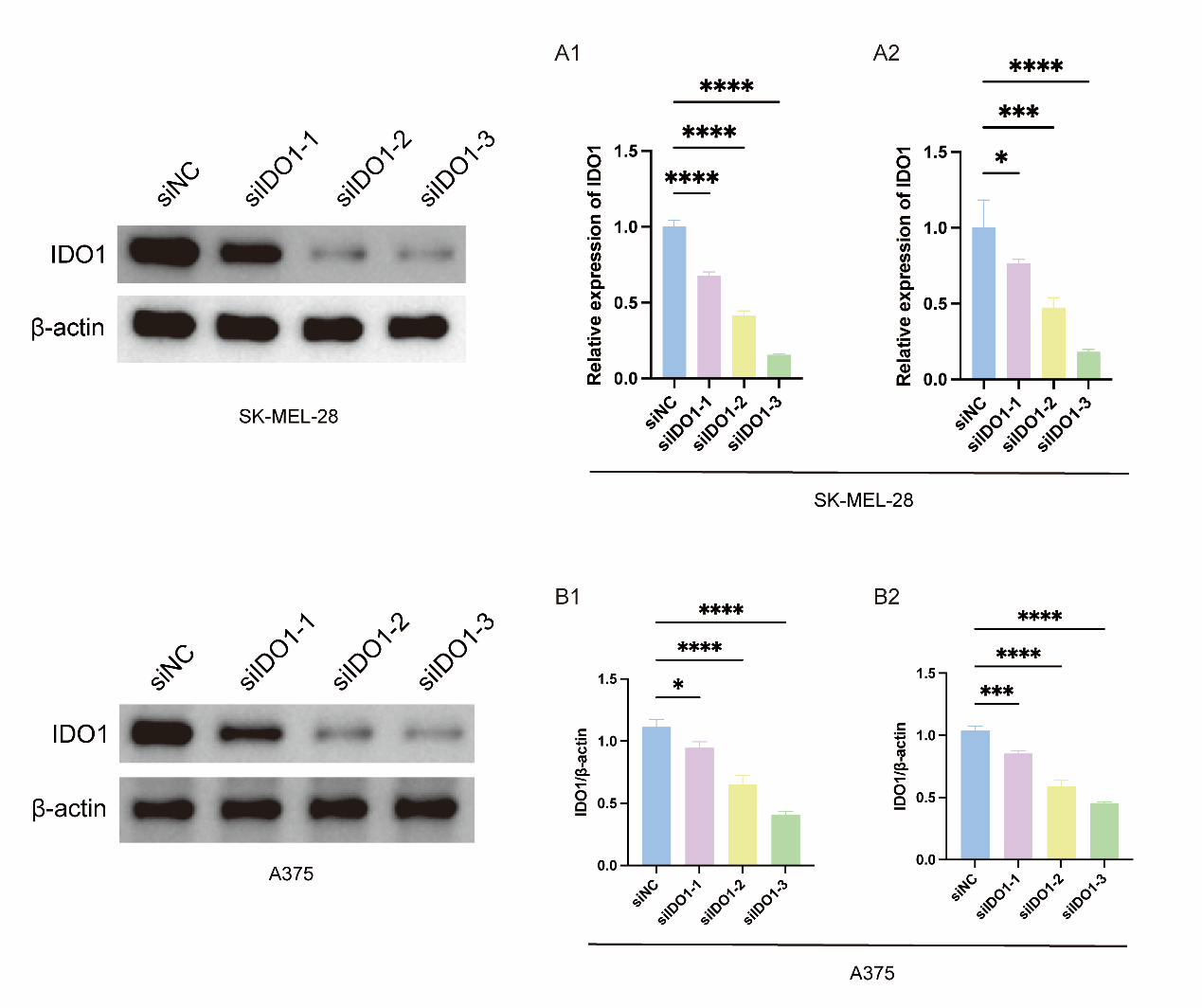


Figure 6. IDO1 knockdown efficiency in SK-MEL-28 and A375 cells. (A, B) Western blot of IDO1 after siRNA transfection. (C–F) Relative IDO1 expression compared with siNC.
