# Supplementary material for: A tryptophan metabolism-related gene signature predicts prognosis and immune features in cutaneous melanoma
Source: Front Immunol. 2026 May 19;17:1806319. doi: 10.3389/fimmu.2026.1806319 (PMC13226596; doi:10.3389/fimmu.2026.1806319)
Supplement: Supplementary file 2 [file Table1.docx]

Table 1. Sequences of siRNA.

| siRNA | Sequence（5'-3'） |
| --- | --- |
| si-NC | UUCUCCGAACGUGUCACGUTT |
| si-IDO1-1 | GGGCUUCUUCCUCGUCUCUTT |
| si-IDO1-2 | CCATCTGCAAATCGTGACTAA |
| si-IDO1-3 | GCAAUAUUGCUGUUCCCUATT |

Table 2. Primer sequences used for quantitative real-time PCR.

| Primer | Sequence（5'-3'） | Product（bp） |
| --- | --- | --- |
| HADHA-F | GAGGAGGACTTGAGGTTGCC | 147 |
| HADHA-R | CAGGCACACCCACCATTTTG |  |
| STAT1-F1 | TGTTATGGGACCGCACCTTC | 128 |
| STAT1-R1 | AGTGAACTGGACCCCTGTCT |  |
| CAT-F | AGGGGCCTTTGGCTACTTTG | 127 |
| CAT-R | ACCCGATTCTCCAGCAACAG |  |
| GOT2-F | GATAAGGATGCCTGGGCTGT | 107 |
| GOT2-R | GCTCCTACACGCTCACCATA |  |
| IDO1-F | GAAAGGCAACCCCCAGCTAT | 167 |
| IDO1-R | GGAGGAACTGAGCAGCATGT |  |
| hGAPDH-F1 | TTCGTCATGGGTGTGAACCA | 170 |
| hGAPDH-R1 | GTCTTCTGGGTGGCAGTGAT |  |

Table 3. Antibody (western blot).

| Antibody | Source | RRID |
| --- | --- | --- |
| HADHA | Proteintech | 10758-1-AP |
| STAT1 | Proteintech | 66545-1-Ig |
| CAT | Proteintech | 21260-1-AP |
| GOT2 | Proteintech | 14800-1-AP |
| IDO1 | Proteintech | 66528-1-Ig |
| β-actin | Proteintech | 66009-1-Ig |
